# Growing up during a public health crisis: a qualitative study of Born in Bradford early adolescents during Covid-19

**DOI:** 10.1186/s40359-022-00851-3

**Published:** 2022-05-31

**Authors:** Bridget Lockyer, Charlotte Endacott, Josie Dickerson, Laura Sheard

**Affiliations:** 1 Bradford Institute for Health Research, Bradford Teaching Hospitals NHS Foundation Trust, Bradford Royal Infirmary, Duckworth Lane, Bradford, BD9 6RJ UK; 2 Department of Health Sciences, University of York, Heslington, York, YO10 5DD UK

**Keywords:** Covid-19, Adolescence, Mental Wellbeing, Bradford

## Abstract

**Background:**

Whilst children and young people have not often been at forefront of the immediate Covid-19 pandemic health response there has been concern about the indirect consequences of Covid-19 on children’s physical and mental health and what the effect of the pandemic will be throughout their lifetimes. Early adolescence is a time of transition and reorientation. This study considers the impact of the first UK Covid-19 lockdown on early adolescents.

**Methods:**

The study topic was identified through a consultation process which aimed to provide appropriate evidence to local decision makers in Bradford, UK and plan for future interventions. A group of children and their parents from the longitudinal Born in Bradford (BiB) cohort study were randomly selected and then purposively sampled by ethnicity, age, sex and deprivation. The BiB cohort is made up of 13,776 children and their families and were recruited at Bradford Royal Infirmary between 2007 and 2011. 41 interviews (with 20 families: 20 parents and 21 children) were carried out between August and September 2020. Interview data was analysed using reflexive thematic analysis.

**Results:**

The transitional age of the children interviewed had an important influence on their experience Covid-19 and the first UK lockdown. Their age combined with lockdown and school closures meant that they missed out on key learning and social opportunities at a crucial time in their lives. Covid-19 and lockdown also disrupted their daily mental wellbeing and led to increased anxiety, lethargy and low moods, during a period of personal change and social transition.

**Conclusion:**

For children at the start of their adolescence undergoing change and formation, the experiences and feelings Covid-19 has set in motion will likely have an impact on their mental and cognitive functioning as they develop further. It is important to acknowledge these early adolescent experiences and continue to monitor and provide targeted support to this group of young people.

## Background

Children and young people have not often been at forefront of the immediate Covid-19 pandemic health response because they have generally been at less risk of severe symptoms and death compared to older adults [1, 2]. However, since the crisis developed there has been concern about the indirect consequences of Covid-19 on children’s physical and mental health and what the effect of the pandemic will be throughout their lifetimes [3, 4]. The impact of rapid and considerable changes to the daily lives of children across the world and of all ages as a result of lockdown(s), school closures and social distancing has been explored in a number of studies [5–11]. This research has helped us to record and understand children’s reactions to the pandemic, including increased feelings of loneliness, low mood, social isolation and higher rates of depression, anxiety and post–traumatic stress disorder, as well as exploring particular vulnerabilities and protective factors [4, 6, 12]. There is emerging evidence that the pandemic will increase health and social inequalities, with the most vulnerable children experiencing more negative impacts on their mental and physical health, child abuse and maltreatment [13].

The adolescent years, which the World Health Organization defines as the ages between 10 and 19, involves significant biological and social transitions [14]. The hormonal, social and psychological changes adolescents experience can lead to changes in mood and behaviour. During this period, adolescents are more likely than adults to experience negative emotional states and their emotional responses tend to be more intense, variable and extreme [15]. It is also a period of significant social reorientation that involves the seeking of social independence from parents/caregivers, renegotiation of privacy boundaries and further integration with and influence of peers [16–18]. Social context is key to this social reorientation, the onset and consequences of puberty mediated by the influences of family, peers and school [19–21]. During this time adolescents start to align their behaviour with the norms of their peer group or the group they wish to belong to [22]. Learning environments can have a significant influence on development outcomes, helping to foster social connectedness, a sense of belonging and interpersonal support from peers and teachers [23–25]. Early adolescence is a particularly vulnerable time, not only because it is the start of a period of these significant hormonal, social and psychological changes but because it also incorporates the transition from primary to secondary school. This transition fundamentally alters the social environment of children, as moving to different schools, different classrooms and making new friends, puts existing primary school friendships at risk [26, 27]. Questions of belonging and social acceptance inevitably arise during this period of social and educational change, and can lead to lower self-esteem and academic achievement and contribute to increased rates of anxiety and depression [27–29]. Since March 2020, early adolescents in the UK have had their lives dramatically altered, impacting on their experience of this transition and reorientation.

Covid-19 was declared a pandemic in March 2020 [30]. Across the world, strict lockdown restrictions came into place shortly after to help reduce the spread of Covid-19. A significant restriction for children was school closures; 190 countries stopped in person teaching, affecting 90% of the world student population [31]. Many schools closed quickly and for the foreseeable future. The UK government announced the first lockdown in the UK on 23rd March 2020, ordering people to “stay at home” and only leave their homes for essential shopping, one form of exercise per day, medical need or care for vulnerable people and travelling to and from essential work. In England, schools stopped in person teaching on 23rd March 2020, except for the children of key workers or children identified as vulnerable [32]. A phased return of school students started in June 2020, but most children did not return until September 2020. At the time of writing, there had been further school closures between January-March 2021, and since September 2020, many children have frequently missed school due to students or school staff members testing positive for Covid-19.

The lockdowns and school closures have meant families have experienced significant disruptions to their routines. These include parents becoming unemployed, being put on furlough or working from home more, home-schooling and the moving between homes for children of separated parents, in addition to separation from wider family and friends and learning social distancing [5]. The move to home-schooling meant families had to navigate a new dynamic within the home, increasing parents’ levels of stress, worry, social isolation, and domestic conflict [33, 34]. Increased tension in the home around new home, school and work pressures and the anxiety associated with the pandemic also impacted on children’s mood [33, 35, 36]. In addition, both parents and children experienced health anxiety around Covid-19, as media reported mounting case and death numbers each day [34, 37].

Previous studies have documented the impact of Covid-19 on children through large scale surveys [5, 7, 9, 11, 38, 39]. some of which included open ended questions which children themselves responded to [8], In contrast, there have been few studies which have conducted interviews with children, and these have been with older adolescents [40] or joint interviews with children from a wide range of ages and their parents [6]. O’Sullivan et al. [6] found that Covid-19 public health measures had adverse implications on children and adolescents’ mental health, particularly for children with existing developmental disorders. Their study however relied mainly on parental observations. Using Reinart method analysis, Idoiaga Mondragon et al. [8] were able to identify the main ideas held by children regarding the lockdown, revealing children’s starkly negative emotional responses to the lockdown. They included open text responses written by children which were particularly resonant and enlightening. We hope we have been able to build on these narratives within our study through the use of interviews, which enable the researcher to encourage participants to explain and clarify meaning.

Our study, which took place concurrently with the above studies, took a more specific focus, bringing attention to the interaction between the experience of early adolescence and the experience of living through the Covid-19 pandemic. Being part of the Born in Bradford (BiB) longitudinal birth cohort study meant we had built up trust with children and their families for years, enabling us to access the experiences of younger adolescents (aged 10–13). Through in depth qualitative interviews with both children and their parents, we set out to explore the particular experience of undergoing a significant life change within an unprecedented world change. In addition, we examine what these changes and pressures meant for children at the beginning of their adolescence within an area of high deprivation, ethnic diversity and persistently high Covid-19 rates. We hope this can help acknowledge the complexities of this experience, identify vulnerabilities and inform ways to better support this generation of emerging young people.

## Methods

### Study design

This descriptive, inductive qualitative study was completed as part of a larger mixed‐method, longitudinal research study which was developed to provide actionable intelligence to local decision makers in Bradford during Covid-19. We used community and stakeholder consultation to identify priority topics for research described in Box 1 [41].

**Box 1:** Patient and Public Involvement (PPI) and stakeholder consultation process to identify critical topics

In March 2020, Bradford’s Covid-19 Scientific Advisory Group was formed to support policy and decision makers in Bradford and the UK to deliver an effective urgent response and to better understand the wider societal impacts of Covid-19. As part of these aims, a rapid community and stakeholder engagement process was used to identify priority topics important to both citizens in Bradford and local decision makers. This process took place in April 2020 via the following engagement activities:	
1. The first author spoke to nine members of Bradford’s District Gold Command (established in response to the Covid-19 emergency). These were brief 15–20 min phone calls to assess their top Covid-19 priorities	
2. Analysis of the first 350 free text responses to the Born in Bradford Covid-19 adult questionnaire. This survey was undertaken during the initial weeks of first lockdown to assess what the main concerns were for parents in the district	
3. Community researchers collected soft intelligence (comprised of informal phone conversations asking about current community concerns around Covid-19) from 13 people considered influential within diverse community settings	
From the above, three priority topic areas for qualitative work were identified: (1) “health beliefs” encompassed: access to healthcare services, experiences of Covid-19, sources of health information and the spread of misinformation, (2) adolescent mental well-being during the lockdown which is the subject of this paper and (3) people living in poverty before Covid-19. Data was collected and analysed on all three topics. The intelligence and evidence obtained from investigating these priority topics was rapidly shared at a local level and influenced Bradford’s public health response [42]	

This consultation process revealed concerns about how children would be affected by being unable to attend school and the impact of lockdown and social distancing on their social and mental wellbeing. These concerns seemed to centre on school-aged children and in particular adolescents. Research on this particular participant group was deemed critical in order to respond quickly to their needs and make recommendations for their futures. Whilst we had some indication of children’s experiences through the free-text responses in the Born in Bradford Covid-19 surveys, it was decided that interviews with children and parents were needed to understand their experiences and reactions in greater depth. Soft intelligence work and further analysis of both the adult and children’s Phase 1 Covid-19 surveys continued throughout the development of this study between May and August 2020, and helped shape the interview questions.

### Study setting and timing

The BiB research programme was in a unique position to be able to study the impact of the Covid-19 response on a key vulnerable population: primary and/or secondary school aged children living in a highly deprived and ethnically diverse city. In response to high rates of childhood ill-health in Bradford, the Born in Bradford (BiB) longitudinal cohort study was established in 2007 [43]. Its purpose was to examine the genetic, environmental, behavioural and social factors that influence the health and development of children in the city, from the perinatal period, during childhood and into their adult years. All mothers who gave birth at Bradford Royal Infirmary between 2007 and 2011 were invited to take part, which resulted in a cohort of 13,776 children and their families. Mothers recruited into the study provided a rich dataset through detailed questionnaire data, measurements and samples. Mothers also consented to the linkage of routine data health and education data concerning themselves and their child. Recent follow-ups of our cohort participants means that we have a detailed understanding of the physical and mental health, social, and economic circumstances of BiB families since index pregnancies/births, including data collected in the recent ‘pre-pandemic’ and ‘pre-lockdown’ period (2016–March 2020) [41].

The interviews took place in August and September 2020, just before the majority of the children returned to school. Since March 2020, Bradford had experienced a relatively high number of Covid-19 cases compared with the rest of the UK, and since July 2020 had been under stricter local lockdown measures which remained in place until the introduction of the tier system in October 2020 where Bradford was placed under the strictest tier [44]. These restrictions included no mixing of households indoors, a ‘rule of six’ outdoors and no travelling in or out of the area. High rates of Covid-19 in areas like Bradford are likely to be due to greater deprivation, high population density and a higher than average number of multi-generational households [45].

### Sampling and data collection

Due to the age of the BiB cohort, we focused on families with children aged 10–13, choosing to interview both parents and children as a way to explore adolescent mental wellbeing from different perspectives [see 46]. Our sample started with 100 families (parents and children) who had both participated in the recent BiB Covid-19 adult and child surveys, split into two groups. The first group (randomly selected from a total of 490) was a sample of 50 children (and their corresponding parent) who reported moderate to low mental wellbeing on the Revised Child Anxiety and Depression Scale (RCADS) in their survey and the second group (randomly selected from a total of 306) was a sample of 50 children (and their corresponding parent) who reported medium to high mental wellbeing on this scale. We decided to exclude children who reported low to very low wellbeing on the RCADS because we felt it would not be ethical to contact these families when we could only offer limited support and signposting at the time due to social distancing. In addition, this group was only 22 children out of a total of 818. This group of 100 were then purposefully sampled by ethnicity, age of child, sex and deprivation (using the index of multiple deprivation) to make the small sample as diverse as possible. We also took into account the parent responses and whether they were concerned about their children’s’ mental wellbeing, and ensured we included those who did indicate it was a concern and those who did not [46]. The sample of 100 was reduced to 50 families this way, with the aim to conduct interviews with around 20 families. Recruitment took place via a letter with information about the study and notice that they might receive a follow up phone call to ask if they wished to participate. 39 families were contacted via phone and asked to participate, 5 declined to take part, 4 did not answer the phone, 10 initially agreed but did not answer follow up calls. Purposive sampling continued throughout the recruitment process to ensure the sample retained its original diversity in ethnicity, age and sex of child and deprivation. This impacted on the order families received follow up calls and eleven families were never contacted by phone as we had reached our target of 20 families. Overall, the first author conducted interviews 41 interviews (with 20 families: 20 parents and 21 children).

The sample of interviewed children consisted of 9 boys and 12 girls aged 10–13, with the majority being 11 or 12 years old. In terms of ethnic group, they were White British (9), Pakistani heritage (7) and other (4) (the label of ‘other’ included children of African and Eastern European heritage) [46]. The index of multiple deprivation associated with where they lived ranged the full spectrum from 1–8, but most families where clustered in the lower numbers (higher deprivation) (see Table 1 for participant demographics). Although it was up to each family to decide, parent interviews were mostly conducted with mothers and only one was with a father. This is because the children’s mothers are usually the primary contact within the larger BiB study, as mothers were recruited during pregnancy and birth.

**Table 1:** Participant demographics

Family no.	Sex of BiB child	Age of BiB child on 31 March 2020	Ethnicity	IMD	Parent	Non-BiB child interviewed
*Medium to high mental wellbeing using RCADS*						
Family 1	Female	10	Other	1	Mum	
Family 5	**Male**	**11**	Pakistani	3	Mum	
Family 7	Male	12	White British	2	Mum	
Family 8	Male	12	White British	5	Mum	Female aged 10
Family 9	Female	12	White British	4	Mum	
Family 10	Female	11	Pakistani	4	Mum	
Family 12	Male	11	White British	4	Mum	
Family 13	Female	12	Pakistani	3	Dad	
Family 14	Male	11	Pakistani	3	Mum	
Family 15	Female	12	White British	8	Mum	
Family 19	Male	11	Other	6	Mum	
*Moderate to low mental wellbeing using RCADS*						
Family 2	Female	11	Pakistani	1	Mum	
Family 3	Female	11	White British	1	Mum	
Family 4	Female	11	White British	1	Mum	
Family 6	Male	12	Other	1	Mum	
Family 11	Female	11	White British	2	Mum	
Family 16	**Female**	**10**	White British	6	Mum	Female aged 10
Family 17	Male	11	Pakistani	1	Mum	
Family 18	Male	12	Pakistani	2	Mum	Male aged 10
Family 20	Female	12	Other	5	Mum	

Bold indicates that the sampled BiB cohort child was not interviewed. In Family 5, the child did not want to be interviewed, and in Family 16, her sibling was interviewed instead

Consent was given verbally by both the parent and child prior to each interview. Due to social distancing measures, the interviews were conducted over the phone or via video call, whichever was the family’s preference. Phone was preferred. For the interview with children, the parent and child were given the option of having the parent present or not, or having a sibling present. The majority of the parent interviews took place whilst the child was out of the room, but the majority of the child interviews took place whilst the parents were around, not necessarily in the room but within hearing range. We understand that this may have made some children more inhibited, particularly as they were approaching an age where they might want to be more private. However, as they were taking part in a virtual interview with someone they had not met before, it was a priority for the research team to ensure children and parents felt safe and comfortable.

### Interview questioning

Through the consultation process we identified important areas to explore such as changes to daily routine, how they felt about school/schoolwork in this period, what activities they were/were not able to do. Topic guides for the child and parent interviews were developed as a flexible document to allow participants to voice what they considered to be important. We paid particular attention to making sure questions to children were tangible and focused on the present. For example, instead of asking ‘tell me about your daily routine during lockdown’ we asked ‘can you tell me a bit about what you did today?’, later asking whether that was a typical lockdown day. Interviews were piloted (4 child, 3 parent) and changes made to improve questioning, especially for the child interviews. The pilot interviews were not recorded and the data has not been included in the analysis.

### Analysis

For the analysis, we used the principles of reflexive thematic analysis [47]. Data analysis was begun by the first author after each interview through the writing of detailed field notes, which helped to identify and organise initial interpretations. Transcripts of each interview were then read inductively multiple times to encourage familiarity with the data set. Nine interviews from four families were selected by the first author because they were considered representative of the interviews in terms of general content and they also were a mixed group in terms of ethnicity and sex of the child. They were analysed independently by the first and last authors and by another experienced member of the research team, who came together to identify commonalities and patterns in the responses and discuss how these could be ordered into loose themes. These were then used and refined by the first author to analyse the remaining interviews. However, because we took a reflexive analytic approach, this process was an ongoing conversation between the first and last author to discuss, question and query the assumptions we were making whilst interpreting and coding the data. Our approach was not to seek full consensus in our interpretations, but to develop a richer and more nuanced reading of the data [47]. This continued during a secondary analysis of the whole data set by the second author at a later time point, where new interpretations and meanings were generated and incorporated. Although the RCADS scores from the survey were used to create the sample, they were not analysed through this lens as there were no discernible differences between the two groups.

## Findings

We found that the transitional age of the children we interviewed had an important influence on their experience of Covid-19 and the first UK lockdown. Their age combined with lockdown meant that they missed out on key learning and social opportunities at a crucial time in their lives. Covid-19 and lockdown also disrupted their daily mental wellbeing, during a period of personal change and transition.

Based on our analysis of the data and the most prominent themes identified, we are presenting our findings under four headings: Anxiety about Covid-19; Boredom and Lethargy; Disengagement from School and Reduced Social Contact.

### Anxiety about Covid-19

Both parents and children indicated that many children were experiencing relatively high levels of worry around the potential health impacts of Covid-19. Some parents described their child as too anxious to leave their homes during the initial lockdown (March-June 2020), even for permitted exercise:

*She wouldn’t even take the dog out, it seemed to really affect her, she was quite scared. And obviously she’s at that age now where she’s got a TV upstairs and she can read, so you know, she’s reading about the coronavirus every day and seeing things, so it did scare her at the beginning and she didn’t, I don’t think she left the house for about eight weeks. (Family 11, Parent)*

This child discussed her own anxiety, and how she had been feeling a bit better since the number of Covid-19 deaths had decreased:

*Well at the start I was like quite worried because I was like worrying that maybe like I’d get it because I just, I didn’t want to go out as much. Kind of like worrying about it but like, when they like started to lower down and there were less deaths…yeah. (Family 11, Child)*

Parents attributed their children’s’ increased anxiety to the constant news cycle about Covid-19 and what their children saw on social media platforms such as YouTube and Instagram:

*He’s kind of, you know, nervous because he keeps hearing this many or how many people died, these people, you know, how many people are positive for this virus and this and this. So first, you know, for few weeks he was keeping looking at those news, everybody every day, you know, telling me, telling me. Then I told him “no, stop looking at this news, yeah, because it’s too much for you”. Yeah, because he was taking in that, you know, then he was worried, he was saying “I’m not going outside” first. (Family 14, Parent)*

Reassuring their children was challenging, because parents also felt unable to make sense of the news and so much was unknown at the time. Some decided that whilst they could not completely control what their children saw or heard, they would stop watching the news as a family and encourage their children to stay away from Covid-19 related stories.

Children reported feeling worried about the personal health impacts of getting Covid-19 but often they were more concerned about their families, particularly if they felt their parents and grandparents were medically vulnerable:

*I was a bit worried about my mum because they had cancer, I think, three years ago, or something. And I was worried that it was easier for them to get coronavirus, so I was really worried for my mum. (Family 17, Child)*

*I just didn’t really know what would happen, because when you just started hearing about Covid-19, I was always worried, will it come to Bradford, will I get it and pass it to my dad, because I’d heard that kids don’t get it as bad as older people. Because I was like, oh, can I pass it to my parents or my nana and will they, summat happen? So I was always scared of that. And then when it did come to Bradford I got really worried. But my parents helped me get through that. (Family 20, Child)*

Both parents and children discussed how they talked through these anxieties, and parents played an important role in comforting and reassuring their children. They became less worried as lockdown measures lifted and the numbers of cases and deaths started to decrease, although at first children were nervous about being in public places. Parents described their children’s vigilance about mask-wearing, social distancing and hand-washing, although this was sometimes taken to the extreme:

*I think that were the major thing, the hygiene thing, they got to the point where they were making, they were washing their hands raw. And I think they were frightened, I know they were frightened for their dad because he’s a bus driver and obviously he’s in his late 50s now and he, I think they were frightened for their dad. (Family 20, Parent)*

This intense period seemed to leave some children with a sense of uncertainty and trepidation what the future held:

*Sometimes I was feeling a bit like anxious because I didn’t know what would happen again, mm. Like because there was like one or two points during lockdown I felt like what would happen? Questioning the future. (Family 14, Child)*

### Boredom and lethargy

As well as experiencing anxiety, children were also facing boredom. Many of the children described lockdown as boring, especially as time had gone on. Some of them had very active lives before lockdown, attending multiple extra-curricular activities such as sports clubs, youth clubs and activities related to their church or mosque. The majority of these had stopped completely, with some carrying on virtually. Whilst most had appreciated a bit of a rest at first they started to feel ‘cooped up inside’ (Family 10, Child) and that their days were repetitive:

*[Being at home felt] amazing but now I get bored a bit and now I’ve not really to do… if I do go back to school it might give me something to do but I don’t know what’s really happening. (Family 12, Child)*

*He was saying “it’s boring, boring”, every day he used to wake up, “oh it’s boring, we can’t go anywhere, so we can’t do anything”. (Family 14, Parent)*

Having no ‘reason to get up in the morning’ was causing some of the children to sleep in and/or get up quite late. Their bedtimes had become more relaxed since not going to school and so they were often getting up a lot later than they usually would, sometimes after midday and in the most extreme example, 2 pm. One child explained why he was getting up a lot later:

*[On] a day like this, a lockdown day, I don’t need to get up so like I just kind of lie asleep through the entire thing. (Family 18, Child)*

As discussed by many of the parents, the majority of the children interviewed were reaching adolescence and were perhaps starting to need or want more sleep and the relative freedom of lockdown had given them more opportunity for this. However, some parents were troubled by their children’s lack of routine and structure in their day which meant they were spending a lot of time alone watching videos or playing computer games on a TV or tablet. Even when they were able to go out and do a few more things, some children had fallen into lethargy:

*No he hasn’t been [out in public] but when I’ve asked him to go with me somewhere you know, just to get him out the house, ‘no, I’ll stay here’, I don’t think that’s because he’s worried about the virus, actually I think that’s because he just wants to stay indoors, you know, so…. it’s just the iPad the whole day. (Family 5, Parent)*

During lockdown, children often had to entertain themselves, because parents were very busy working from home or working as key workers outside of the home. Some parents were worried about how they could get their children back into a routine when school started and whether they would struggle with concentration. This boredom combined with anxiety often meant children were more irritable than usual, less tolerant of their siblings, experiencing mood swings or low moods. Parents reported that they were unsure whether this could be attributed to their age or to the impact of lockdown.

*She did cheerleading like four times a week so for that to stop suddenly as well has had a major…as well because everything we’ve known has just stopped, do you know, everything from like going to school and going straight out after school, to being at home, it has, it stopped for her, her whole little life has changed dramatically. So the mood being down from being sociable to being at home all the time. (Family 4, Parent)*

### Disengagement from school

Whilst a lot of children were unhappy that they were not able to go out and enjoy their hobbies and activities or go on day trips and holidays, a major cause of their boredom was not being able to attend school. They often described school as quite stressful, particularly if they had recently made the transition to secondary school and time off from getting up early, stern teachers, difficult social situations and doing homework was welcome at first. After a while however they became fed up with the monotony and most were quite eager to return to school:

*Well like in June we go to school like 2 days, like was good because obviously like them 2 days were like alright, but now obviously because I’ve stayed here for like 4/5 months now so obviously it gets a bit more boring. (Family 2, Child)*

Some parents had adopted a strict home-school regime and others were more relaxed. This was often dependent on whether their schools were offering contact hours, guidance and feedback, whether the parents were working or not and whether there were younger children in the home who needed more help. Parents would describe how arguments and tension were often triggered by home-schooling, as both parents and children had to adapt into different roles, of teacher and student, within a home environment:

*They lose their routine like you know, [at] the school [they] sit down with other friends having a proper teacher with them and I think for them it was just you know, staying at home, ‘what’s the point of education?’ at the time. (Family 19, Parent)*

Children would often become frustrated when they felt unable to do the work that had been set:

*He has been quite anxious and stressed, I’d say we did have a lot of tears, a lot of kind of storming off saying he couldn’t do his work and that kind of thing. (Family 15, Parent)*

Parents were concerned their children would fall behind and struggle to get back to their previous level of ability and concentration:

*She’d lost her focus, her enthusiasm and she didn’t then want to do even a simple book review, so doing a project on a country was quite good but again, once it was done, that was it, interest was lost once again. So she’s definitely suffered. (Family 16, Parent)*

A few of the children were also concerned about the impact of lockdown on their intellectual abilities:

*It feels weird because it’s been such a long time that I haven’t been to school, is that sometimes I can’t even like speak properly without messing up or, you know, doing something. I realise that I’ve thought a bit slower and I’m not as like, you know, sharp as I was, as I was in school... I was a really good student before. I feel like quarantine made me a little less smart, in a sense that I’ve not been as sharp as I used to be, used to. And I’ve just kind of been lacking in just intelligence, really. (Family 18, Child)*

Many of the children interviewed had just started secondary school or were just about to, so were in a time of transition. For those who had just started, they were still getting used to a larger school and some had experienced issues adapting to this new environment. These children were usually happy to not be attending school but their parents were worried that the lockdown would have a further destabilising effect. Those who were just about to start secondary school were sad they did not get the expected end of primary school experience or get to say goodbye properly to their teachers and friends going to other schools.

*I’m sad because like I don’t get to say goodbye to my teachers. Yeah, we got split… like we needed to social distance say goodbye, like we waved and like said bye and stuff. (Family 1, Child)*

*It’s been quite hard not being able to see [my friends] because like sometimes I get upset that, sometimes like I may not like see any of them again because a lot of them are going to [a different school].(Family 11, Child)*

They had also not been able to visit their new school and have any transition sessions and this was causing anxiety for some children and their parents.

### Reduced social contact

For this group of children, school was usually at the centre of their social life and face to face interaction with their friends was their main mode of social contact. Due to their age, some were unable to maintain friendships without in-person contact and many of the younger children did not yet have their own phones and/or social media accounts which vastly reduced their means of independently contacting their friends during lockdown:

*Because normally, even before the lockdown, he’ll only meet his friends in school, their relationship was in school than outside the school so since the lockdown there hasn’t been any contact with friends because he doesn’t contact them, (Family 6, Parent)*

*I just missed hugging [my friends], even when I got to school I couldn’t hug them. I was only able to keep in touch with my best friend because I don’t think Mummy had any other, like any other of my friends emails or anybody, so… (Family 16, Child)*

*She didn’t really, she didn’t speak to anybody on, you know, on her phone, she didn’t talk. So it sort of got cut off, really, pandemic, and then, you know, when we were allowed to meet up, me and her dad were, sort of pushed for her to meet her friend again. But it were sort of only, they only met up twice. (Family 20, Parent)*

Those that were in contact via phone or video calling, social media or texting, often described it as strange compared seeing them in person and that communications could often be misinterpreted or misread:

*You can’t really see them in person. And like sometimes when you text them, they don’t really understand them. (Family 4, Child)*

*It’s just not the same because it’s just not like seeing your friends. You’re talking to their voice, not to them. It’s just different and weird. (Family 8, Child)*

They also discussed finding it awkward and difficult to meet up with friends after so long out of contact:

*‘One of the hardest things to adjust to was socialising, because you hadn’t done it for that long, it was really hard to adjust to’. (Family 8, Child)*

*‘I couldn’t even figure out what to talk about, how to string a sentence together, we was just both sat there, drinking, not knowing what to say’. (Family 9, Child)*

Lack of regular contact meant they felt detached form what was going on their friends’ lives and sometimes they were worried about them:

*I don’t know what they were doing, if my mate’s doing other stuff and it were my mate’s birthday the day after mine so I hadn’t heard owt from him for a while, my mate, he had an accident so he wasn’t able to call me for about a week or so. (Family 10, Child)*

Some children did have contact with their friends via gaming but this was reliant on having access to a games console and good Wi-Fi which was a barrier for some households. Children would often describe trying and failing to video call their friends because their friends had poor Wi-Fi reception.

### Reflections on the findings

We did not find much divergence between the narratives of the children and parents about children’s experiences. Parents were more likely to offer more detail about their children’s behaviours and feelings during this time, and be more specific about certain issues such as the pressures of home schooling. Their responses were in general longer than the children’s, but on the whole they complemented one another. For example, if a parent told us their child had experienced health anxiety during the lockdown, the child would bring this up when they were asked if they had been worried about anything and vice versa if the parent did not identify this issue. One reason for this might be that the parents and children in this study had spent even more time than usual together over the preceding months, and this closeness meant their understanding of their recent experiences were particularly aligned.

Whilst we made efforts to maintain diversity of ethnicity, sex, age, deprivation in our small sample, it was difficult to discern the impact of these individual factors on the experiences of this group of early adolescents. Some differences between families emerged, such as parents’ work situations (working from home, on furlough, working as key worker) or lack of space within their homes to carry out multiple children’s home-schooling alongside parents’ domestic and external work but was difficult to pinpoint how these differences impacted on wellbeing from the narratives offered. We acknowledge and have identified in separate studies on families within this cohort that financial insecurity and ethnicity were important elements on families’ experience of Covid-19 and lockdowns [34, 48]. However, the purpose of this study was not to compare groups but to describe the common experiences of those within the Born in Bradford cohort. We consider some of the impact of Covid-19 on early adolescent wellbeing in general, whilst taking into account that these young people lived in area of high deprivation, ethnic diversity and persistently high Covid-19 rates.

## Discussion

Through in depth interviews, this study has been able to highlight the impact of the Covid-19 pandemic on children at a particular and important time of their lives: early adolescence. We found that their age and the transitions they were undergoing, significantly affected their experience. While many parents tried to shield their children from the Covid-19 news cycle, they were of an age to be aware of the impacts, both personally and for wider society, which led to prominent health anxieties. Boredom and lethargy increased over time and contributed to mood swings. Both children and parents were worried about falling behind with education missing key school milestones (for example, prom or transitions days in high school). Outside of school, these young adolescents found it difficult to maintain social connections with friends, especially if they did not have access to social media or a mobile phone.

Health anxiety featured prominently in the interviews and we argue that their life stage made them particularly vulnerable to these anxieties. Bray et al., [37] found that children as young as seven understood the severity of Covid-19, despite parent/care-givers attempting to shield children from the most distressing news. There is evidence that being exposed, either directly or indirectly, to media videos and images of traumatic events on a daily basis can play a role in causing emotional and psychological distress in young people [49]. Early adolescence is a time when children’s cognitive abilities develop so that they can better understand and make sense of abstract threats such as a pandemic and they start to take ownership over health-relevant behaviours [50]. However it is a very formative and sensitive time in their transitions into adolescence. The children in our study were old enough to independently come across and even seek out Covid-19 information but still needed a lot of reassurance and explanation from their parents in order to make sense of it and reduce anxiety. By early adolescence, children are starting to have a greater ability to understand the real impact of disastrous events on their lives this means they particularly benefit from discussing their emotions, feelings, or doubts with caregivers [49].

Simultaneous to experiencing health anxiety, many children and parents reported increased boredom and lethargy. Again their age and life stage appeared to exacerbate these feelings. Pre-pandemic, many of the children interviewed had still been involved in adult-assisted, structured activities after school or at weekends. A rapid reduction in these activities and the lack of school routine meant many were sleeping later and longer, spending a lot of time on electronic devices and becoming more irritable and moody. In the interviews, parents were unsure whether this change in their children was due to their age and hormonal changes or due to the pandemic. We contend that it was complex interaction of both. We know that around puberty, adolescents tend to become more ‘evening typed’, and start to prefer later bedtimes, and wake times [51]. In addition, their moods become more volatile and they are more inclined to spend more time alone [15, 18]. These changes were described by both children and parents in our study. It appeared that the conditions of the pandemic, the lack of routine, disengagement from school and the opportunity for intensive internet and social media use, made these changes more marked.

Early adolescence also influenced how they experienced home schooling. They were expected by their teachers and parents to be independent learners to a degree, which many struggled with. Parents’ time was divided between key work, homeworking, domestic tasks and helping younger siblings, and when they could give attention, home-schooling was often a point of tension. There were a lot of disparities between the learning support different schools offered, and some found it difficult to access online resources due to household resources. In the Netherlands, Green et al. [52] found that tension and depression was particularly pronounced in younger adolescents during the pandemic and this was strongly linked to experiences of family stress and inequality of opportunity in online home schooling. These struggles and tensions, combined with the boredom and lethargy many felt, meant children appeared to lose confidence in their abilities to learn and focus.

The majority of the children interviewed were in school transition years, either in their last year of primary school or their first years of secondary school. This was a sensitive time, as the younger participants were preparing to adjust to a new environment, friends, teachers, routine and the older ones still in a period of adjustment. Reduced social contact as a result of social distancing measures left many children in our study without regular contact with their friends. Many had not yet reached the age or stage where they managed their own social lives, had their own phones and saw friends outside of school/structured activities. At a time when gaining independence from parents and integration with peers is particularly important, these young people were isolated [16–18].

Since the start of the Covid-19 pandemic, there has been a concern for children’s mental wellbeing and studies have reported increases in loneliness, anxiety, isolation and depression. These studies have largely been quantitative and survey based. Through in depth interviews with both children and their parents, our study has been able to examine the impact Covid-19 and government measures to prevent its spread has had on early adolescents in a city in the North of England with high deprivation levels. This has allowed us to recognise this particular experience and acknowledge the specific vulnerabilities of this age group. The recognition of this experience, awareness of their anxieties, lethargy, learning concerns and social separation, will be important to the development of tailored support to this group as they get older. Their mental wellbeing will need to be continued to be monitored. Our particular concern is the long-lasting effect of health anxiety as excessive and persistent worry is one of the main symptoms of anxiety disorders which are already the most prevalent of the mental disorders in adolescents [53]. Our study has underlined further that treatments will need to be developmentally appropriate, taking into account the specific pandemic experiences and age-related challenges of this group [50].

Our results can also provide some ideas to prepare for a similar event and mitigate issues within this age group. These include: greater universal health and media literacy and increased access to trusted and credible information sources; increased mental support for parents and caregivers during times of crisis, so that they can offer reassurance help to reduce anxiety in their children; a similar standard of distance learning across different schools which help combat limited household resources and parental time pressures and encouraging parents to take a more active role in facilitating their children’s social interaction (e.g. swapping phone numbers with other parents and planning virtual or outside meet-ups).

The interviews for this study took place in what would turn out to be quite early in the lifetime of the pandemic in August and September 2020. In the UK, there have been subsequent waves, lockdowns and school closures. As a result we don’t know what the full impact of Covid-19 has been on early adolescents since, and how they have adapted to continued changes in their routine and different health information. This study was snapshot in time, but is still valuable in helping us understand the initial impact of Covid-19 on this age group, and how it might continue to affect them.

The families in this sample were more deprived on average and families of Pakistani heritage were over-represented compared to the general UK population. Although this could be said to limit generalisability, it incorporates population groups who are less likely to be represented in research studies of this kind.

## Conclusion

Worldwide, the psychological well-being of adolescents around the world has been in decline since 2012 [54]. Numerous studies have indicated that Covid-19 will not improve this situation and there is real concern about what the long-lasting implications will be [3, 4].This study has explored the impact of Covid-19 and subsequent public health measures on children in their early adolescents. This group were and are in a period of transition and formation and they missed out on key social and learning experiences. As a result, the experiences and feelings Covid-19 has set in motion will likely have an impact on their mental and cognitive functioning as they develop further. Crucially, they had felt unsafe and anxious at a sensitive time in their lives when their perceptions of the world were beginning to form independently from the adults around them. It is important to acknowledge these early adolescent experiences and continue to monitor and provide targeted support to this group of young people.

## Data Availability

The dataset collected and analysed during the current study are not publicly available as this could compromise participants’ privacy. The corresponding author can be contacted with questions regarding the dataset.

## Abbreviations

BiB: Born in Bradford
RCADS: Revised Child Anxiety and Depression Scale
IMD: Index of Multiple Deprivation
